# Supplementary material for: High Frequency Production of T Cell-Derived iPSC Clones Capable of Generating Potent Cytotoxic T Cells
Source: Mol Ther Methods Clin Dev. 2019 Dec 24;16:126–35. doi: 10.1016/j.omtm.2019.12.006 (PMC6965501; doi:10.1016/j.omtm.2019.12.006)
Supplement: Document S1. Table S1 and Figures S1–S4 [file mmc1.pdf]

OMTM, Volume *16*

Supplemental Information

High Frequency Production of T Cell-Derived iPSC Clones Capable of Generating Potent Cytotoxic T Cells

Seiji Nagano, Takuya Maeda, Hiroshi Ichise, Soki Kashima, Manami Ohtaka, Mahito Nakanishi, Toshio Kitawaki, Norimitsu Kadowaki, Akifumi Takaori-Kondo, Kyoko Masuda, and Hiroshi Kawamoto

Well #	Sorter count	Manual count after sorting	No. of initial iPSC-like colonies	No. of established iPSC clones
1	1.65×10^5	N.D	4	1
2	4.03×10^5	N.D	8	3
3	3.14×10^5	N.D	0	0
4	0.99×10^5	N.D	3	3
5	2.65×10^5	N.D	5	2
6	1.45×10^5	N.D	1	0
7	3.16×10^5	2.16×10^5	5	1
8	1.41×10^5	0.8×10^5	4	1
9	N.D			
Total	1.84×10^6	1.1×10^6 (estimated)	30	11

Table S1. Efficiencies of establishing T-iPSC clones from MART-1 specific CD8 T cells

Numbers of MART-1$^+$CD8$^+$cells isolated by a cell sorter are shown as a sorter count and a manual count, as well as numbers of iPSC-like colonies initially formed and eventually established as iPSC clones, are displayed for each well in the experiment shown in Figure 1.

A B

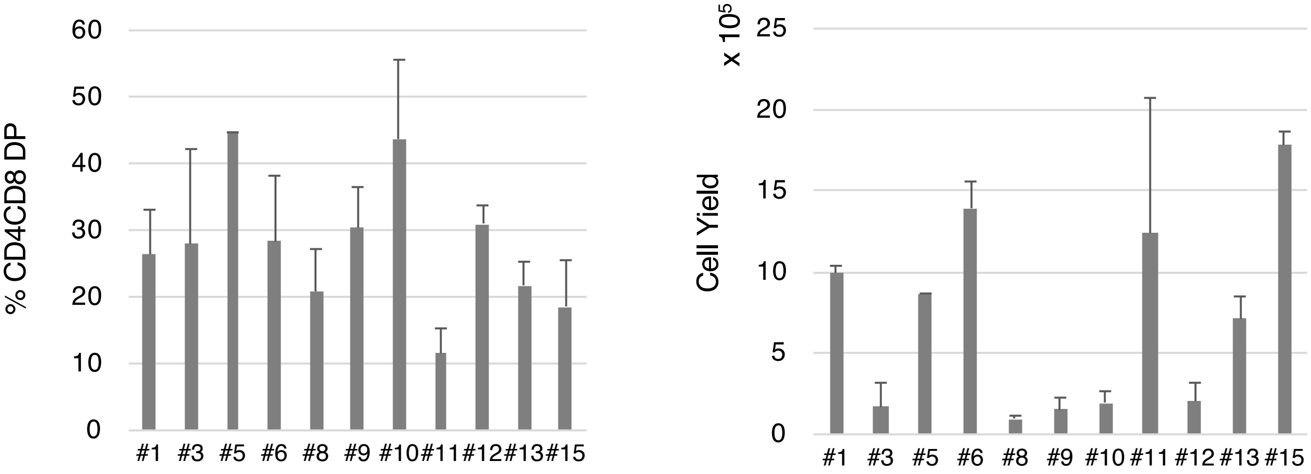

Figure S1. Heterogeneity of monocyte-derived non-T-iPSC clones with regards to T cell generating potential

A) The percentage of $CD4^+CD8^+$DP cells in cells generated from each non-T-iPSC clone. **B)** The total number of cells generated from each clone in one 10 cm dish. Data are shown as averages of two or three independent experiments with error bars representing SEM.

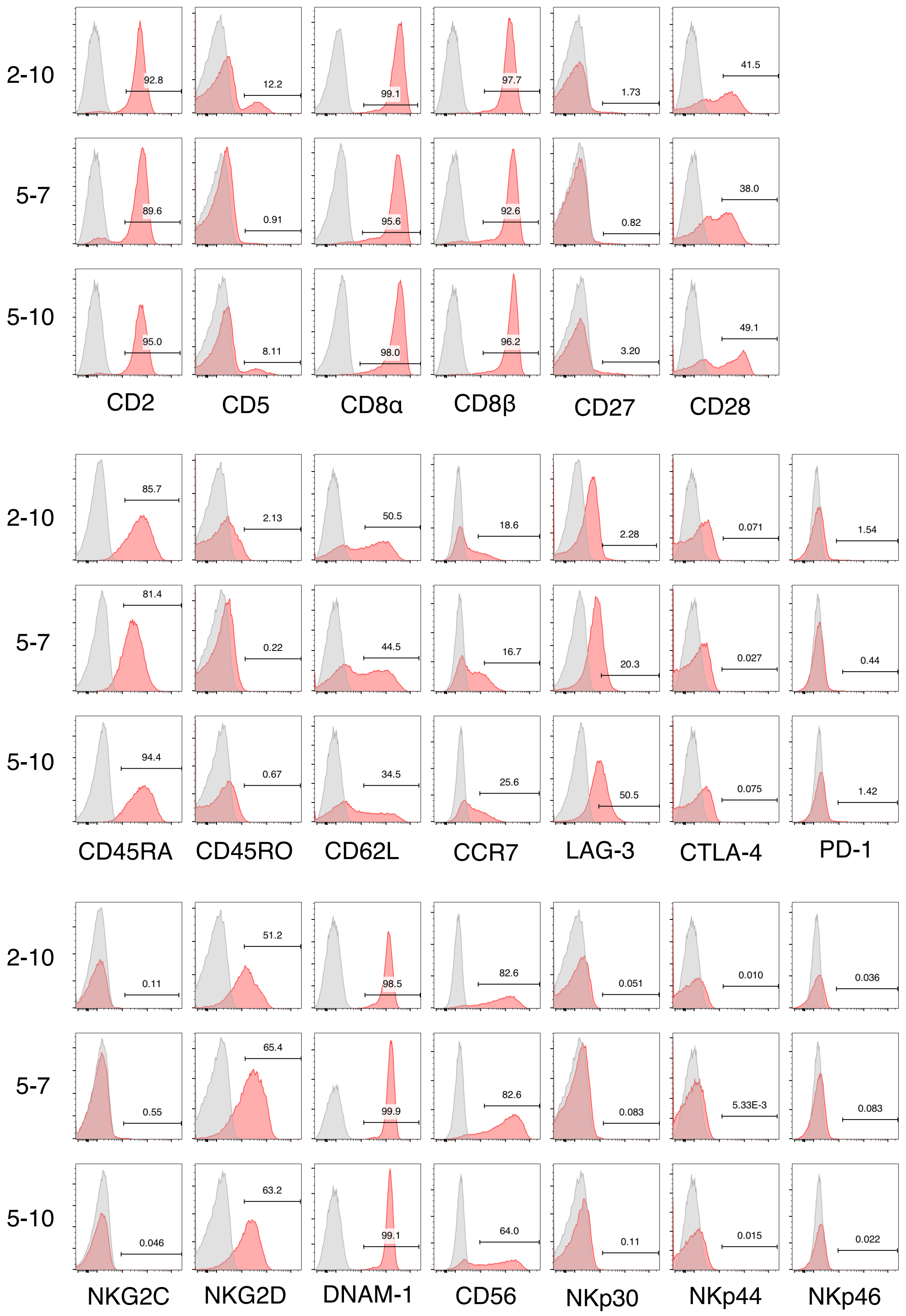

Figure S2. The regenerated CTL clones showed almost identical cell surface phenotypes

Flow cytometric analysis of three regenerated CTL clones representing TCR high (2-10), medium (5-7) and low (5-10) affinity, for the expression of indicated antigens.

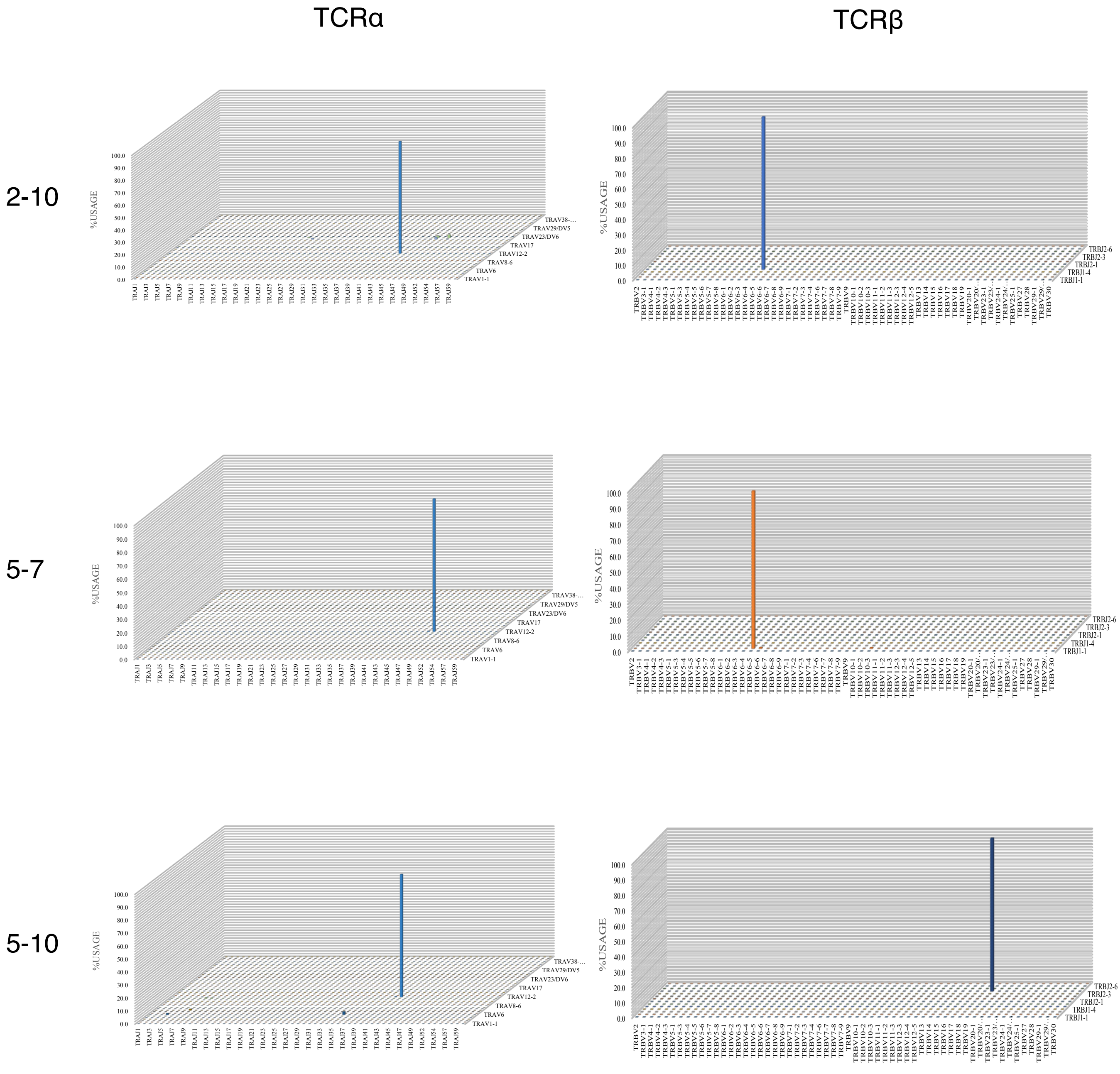

Figure S3. TCR repertoire analysis of regenerated CTLs demonstrates their monoclonality

CTLs regenerated from representative three T-iPSC clones (2-10, 5-7, 5-10) were analyzed for specific CDR3 amino acid sequences, usages of TCRα chain variable (TRAV) and joining (TRAJ) region genes for TCRα, as well as usages of TCRβ chain variable (TRBV) and joining (TRBJ) region genes for TCRβ genes.

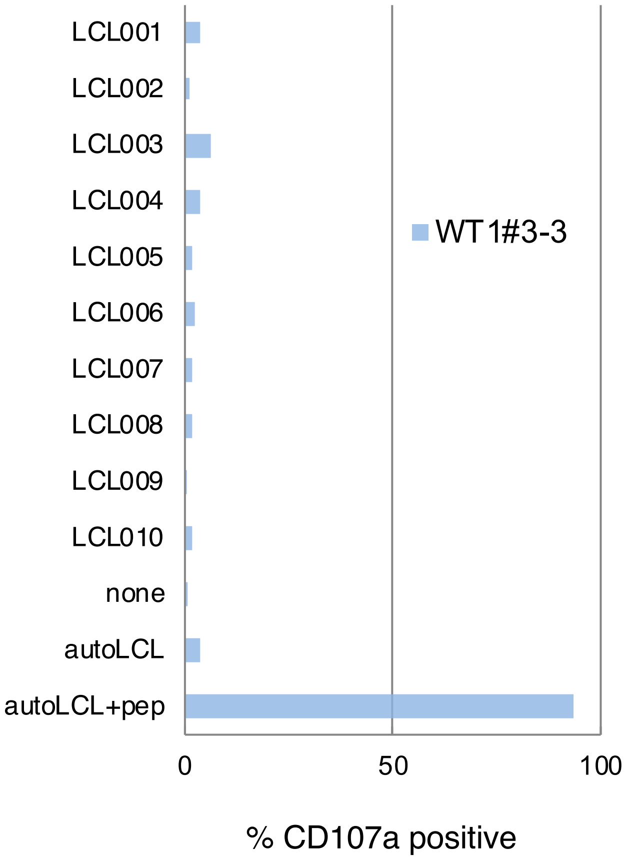

Figure S4. Regenerated CTLs specific for WT1 antigen did not show alloreactivity against ten LCLs

Regenerated CTLs from WT1#3-3 T-iPSCs were tested for allo-reactivity in the same manner as Figure 4. Bars represent the percentage of CD107a positive activated cells. The ratio of CTLs/stimulators was fixed at 1:1. Autologous LCL without peptide pulse was used as a negative control, and autologous LCL pulsed with WT1-peptide$_{235\text{-}243}$ was used as a positive control.
