# Supplementary material for: High Frequency Production of T Cell-Derived iPSC Clones Capable of Generating Potent Cytotoxic T Cells
Source: Mol Ther Methods Clin Dev. 2019 Dec 24;16:126–35. doi: 10.1016/j.omtm.2019.12.006 (PMC6965501; doi:10.1016/j.omtm.2019.12.006)
Supplement: Document S2. Article plus Supplemental Information [file mmc2.pdf]

Molecular Therapy
Methods & Clinical Development
Original Article

High Frequency Production of T Cell-Derived iPSC Clones Capable of Generating Potent Cytotoxic T Cells

Seiji Nagano,[1,2] Takuya Maeda,[1,2] Hiroshi Ichise,[1] Soki Kashima,[1,3] Manami Ohtaka,[4] Mahito Nakanishi,[4] Toshio Kitawaki,[2] Norimitsu Kadowaki,[2] Akifumi Takaori-Kondo,[2] Kyoko Masuda,[1] and Hiroshi Kawamoto[1]

[1]Laboratory of Immunology, Institute for Frontier Life and Medical Sciences, Kyoto University, Kyoto 606-8507, Japan; [2]Department of Hematology and Oncology, Graduate School of Medicine, Kyoto University, Kyoto, Japan; [3]Department of Urology, Akita University Graduate School of Medicine, Akita City, Japan; [4]Biotechnology Research Institute for Drug Discovery, National Institute of Advanced Industrial Science and Technology (AIST), Tsukuba, Ibaraki, Japan

Current adoptive T cell therapies conducted in an autologous setting are costly, time-consuming, and depend on the quality of the patient's T cells, and thus it would be highly beneficial to develop an allogeneic strategy. To this aim, we have developed a method by which cytotoxic T lymphocytes (CTLs) are regenerated from induced pluripotent stem cells that are originally derived from T cells (T-iPSCs). In order to assess the feasibility of this strategy, we investigated the frequency of usable T-iPSC clones in terms of their T cell-generating capability and T cell receptor (TCR) affinity. We first established eight clones of T-iPSCs bearing different MART-1-specific TCRs from a healthy volunteer. Whereas all clones were able to give rise to mature CTLs, cell yield varied greatly, and five clones were considered to be usable. TCR affinity in the regenerated CTLs showed a large variance among the eight clones, but functional avidities measured by cytotoxic activity were almost equivalent among three selected clones representing high, medium, and low TCR affinity. In a total of 50 alloreactivity tests using five CTL clones versus ten target cells, alloreactivity was seen in only three cases. These findings collectively support the feasibility of this T-iPSC strategy.

INTRODUCTION

Currently, adoptive T cell therapy has been mainly conducted in an autologous setting; peripheral blood T cells are collected from a patient and then given back to that patient after *ex vivo* activation, expansion, or genetic manipulation.[1,2] However, such a strategy is costly, time-consuming, and depends on the quality of the patient's T cells, which is frequently suboptimal due to the disease itself or the side effects of concomitant therapies including chemotherapy-induced immunosuppression, and hence can lead to cell therapy failure. To overcome these issues, it would be desirable to develop a strategy where "off-the-shelf" T cells are prepared for use in an allogeneic setting. To this aim, we previously considered a method in which cytotoxic T lymphocytes (CTLs) are cloned and expanded by using induced pluripotent stem cell (iPSC) technology; when iPSCs are produced from antigen-specific T cells (T-iPSCs), rearranged T cell receptor (TCR) genes are inherited by such T-iPSCs and thus the CTLs regenerated from the iPSCs should exhibit the same antigen specificity as the original CTLs.[3] Consistent with this idea, we have succeeded in producing iPSCs from T cells and in regenerating potent tumor antigen-specific CTLs from these T-iPSCs.[4] With these successes, we thought of the idea to use human leukocyte antigen (HLA)-matched donors: i.e., tissue/cells from a donor who has the same HLA allele on both chromosomes (HLA-haplotype homozygous: HLA-homo) can be transplanted to HLA-haplotype heterozygous (HLA-hetero) recipients, expecting that the immunological rejection could be minimal.[5] Thus, we took the following approach: (1) collect T cells from healthy HLA-homo volunteers; (2) expand tumor antigen-specific CD8 T cells from these T cells; (3) produce iPSCs by reprogramming the CD8 T cells; (4) regenerate CTLs from the iPSCs; and (5) inject them into an HLA-hetero cancer patient whose cancer cells express the same tumor antigen.

The above strategy, however, still faces some issues that must be resolved before clinical application: (1) iPSC clones are very heterogeneous in terms of T cell-generating potential,[6] (2) the TCR affinity varies greatly,[7] and (3) use of certain TCRs in an allogeneic setting may cause alloreactivity against the recipient's normal tissue/cells.[8] Due to issues (1) and (2), it is necessary to first produce multiple clones and then stringently select the best one among them. The third issue will require us to test whether regenerated CTLs have alloreactivity against recipient cells before their transfer. If such alloreactivity is seen very frequently, it would be necessary to prepare multiple T-iPSC clones even against a single target antigen. It could be argued that, while the issue (1) should be tested among iPSC clones, the issues (2) and (3) could be tested before producing iPSCs from CTLs. However, it is easier for us to first produce iPSCs and characterize the T cells regenerated from each iPSC clone than to clone CTLs before reprogramming them.

Received 28 October 2019; accepted 10 December 2019;
https://doi.org/10.1016/j.omtm.2019.12.006.

Correspondence: Hiroshi Kawamoto, Laboratory of Immunology, Institute for Frontier Life and Medical Sciences, Kyoto University, 53 Kawahara-cho, Shogoin, Sakyo-ku, Kyoto 606-8507, Japan.
E-mail: kawamoto@infront.kyoto-u.ac.jp

This is an open access article under the CC BY-NC-ND license (http://creativecommons.org/licenses/by-nc-nd/4.0/).

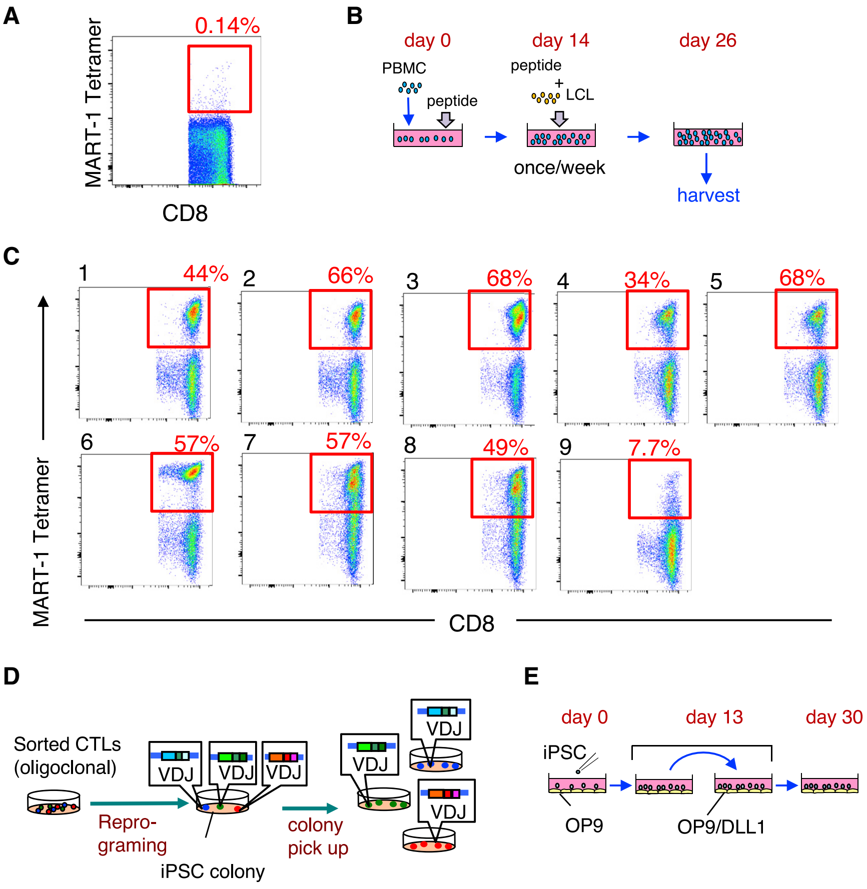

well #		clone #	TCR α Vα	TCR α Jα	TCR β Vβ	TCR β Jβ	TCR β Dβ
1		1-4	12-1*01	45*01	20-1*01	1-1*01	1*01
2	*	2-1	12-2*02	42*01	6-5*01	1-5*01	1*01
	*	2-10	12-2*02	42*01	6-5*01	1-5*01	1*01
	*	2-15	12-2*02	42*01	6-5*01	1-5*01	1*01
4		4-1	12-2*02	5*01	30*01	1-5*01	1*01
	**	4-2	12-2*02	35*01	27*01	2-5*01	1*01
	**	4-3	12-2*02	35*01	27*01	2-5*01	1*01
5		5-7	12-2*02	47*02	6-5*01	1-2*01	1*01
		5-10	12-2*02	41*01	19*01	2-4*01	2*02
7		7-3	12-3*01	45*01	4-1*01	1-1*01	1*01
8		8-6	12-3*01	48*01	5-4*03	2-7*01	1*01

Figure 1. Establishment of Eight MART1-T-iPSC Clones Derived from MART-1-Specific CD8 T Cells

(A) Flow cytometric profile of PBMCs of a healthy HLA-A*02:01 volunteer for MART-1 tetramer versus CD8 expression. (B) Schematic illustration of the procedure for expansion of MART-1-specific CD8 T cells. PBMCs were primed with peptide. From day 14, cells were stimulated using MART-1-peptide-loaded LCLs once per week. (C) Cells harvested after stimulation (day 26) were analyzed for MART-1-tetramer versus CD8 expression by flow cytometry. (D) Schematic illustration of T-iPSC cloning strategy. When multiple colonies were formed in a same well, each colony was individually picked up. For simplicity, only the TCRβ V(D)J rearrangements are depicted. (E) Schematic illustration of the culture method for T cell differentiation from iPSCs. iPSCs were first co-cultured with OP9 stromal cells and then switched to co-culture with OP9/DLL1 cells on day 13. (F) TCRα and β chain V(D)J usage of regenerated T cells from the established MART1-T-iPSC clones. A bar with one and two asterisks indicates that they have the same TCR genes.

In the present study, we addressed these issues and decided to comprehensively evaluate how heterogeneous T-iPSC clones are and to show an accurate estimation of how many clones are required to obtain a good one, by first making multiple clones and testing them. In order to produce multiple clones for this analysis, we selected the melanoma antigen MART-1 as a target, since the frequency of CTLs bearing a MART-1-specific TCR is known to be very high compared to other antigens.[9] We established a total of eight T-iPSCs clones bearing different TCRs specific for MART-1 and examined their heterogeneity in terms of T cell-generating potential and cytotoxicity of the regenerated CTLs, as well as how frequently they show alloreactivity. Based on the results, we estimate that production of eight clones is sufficient to reliably obtain two potent and usable T-iPSC clones.

RESULTS

Establishment of Multiple iPSC Clones from MART-1-Specific CTLs

We first expanded antigen-specific CTLs using peripheral blood mononuclear cells (PBMCs) obtained from a healthy HLA-A*02:01-positive donor. Before expansion, MART-1-specific CD8 T cells, defined as MART-1-tetramer-positive cells, were found at a frequency of around 0.14% of CD8 T cells (Figure 1A). Whole PBMCs were stimulated using the MART-1-peptide$_{26-35(A27L)}$ for 14 days. Then, PBMCs were stimulated once per week using a lymphoblastoid cell line (LCL) loaded with MART-1-peptide as an antigen-presenting cell (Figure 1B). On day 26, MART-1-tetramer-positive CD8 T cell populations were formed in all nine wells used in the expansion culture (Figure 1C). Among these wells, we discarded one of them (#9) in which the MART-1-tetramer-positive population was rather small.

MART-1-specific CD8 T cells in each of the remaining eight wells were separately purified by fluorescence-activated cell sorting (FACS) and stimulated with CD3/CD28 beads. On day 2, these cells were transduced with Yamanaka factors and SV40 large T antigen as

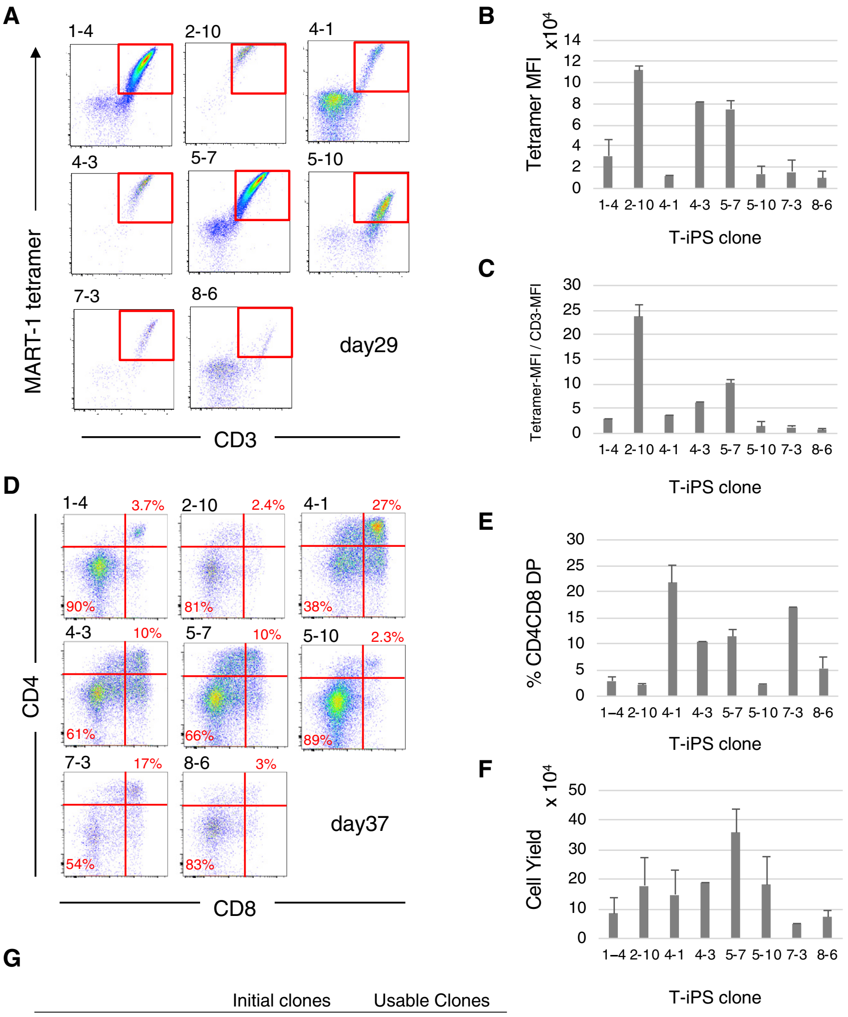

G

	Initial clones	Usable Clones
T-iPSC		
MART-1 (present study)	8	5
WT-1	10	4
non-T-iPSC (monocyte-derived)	11	6
total	29	15

Figure 2. Heterogeneity of MART1-T-iPSC Clones with Regards to TCR Affinity and T Cell-Generating Potential

(A) Flow cytometric profiles of $CD4^{-}CD8^{-}$DN T cells generated from eight T-iPSC clones analyzed on day 29 for MART-1-tetramer binding and CD3 expression. Flow cytometric data are from one experiment representative of two independent differentiation experiments. (B) The tetramer MFI of the generated DN T cells ($CD3^{+}$ gated). (C) The ratio of tetramer MFI versus CD3-MFI of the eight T cell clones ($CD3^{+}$ gated). (D) Flow cytometric profiles of T cells generated on day 37 for the expression of CD4 versus CD8. Flow cytometric data are from one experiment representative of at least three independent differentiation experiments. (E) The percentage of $CD4^{+}CD8^{+}$DP cells in cells generated on day 37 from each T-iPSC clone. (F) The total number of cells generated from each clone in one 10 cm dish. Bar graphs are shown as average of two or three independent experiments with error bars representing SEM. (G) Summary of three experiments evaluating T cell-generating potential of iPSC clones: iPSCs from T cells specific for MART-1 (the present study) or WT1 antigen and iPSCs from monocytes.

previously described.[4] iPSC colonies started to appear from day 11 in seven out of eight wells, and in some wells, multiple colonies appeared. In this case, the colonies were individually picked up, expecting that each colony could represent a different clone in terms of TCR gene sequence (Figure 1D). We thus established a total of 11 iPSC lines from a total of 1.1×10^{6} T cells (Table S1). Hence, reprogramming efficiency was estimated to be around $1/10^{5}$.

To test the clonality of these lines, we induced T lineage cells so that the TCR genes are expressed (Figure 1E). All lines gave rise to T lineage cells, from which cDNAs encoding TCRα and β chains were prepared and sequenced. TRAV12-2 was preferentially used as a Vα gene in the MART-1-specific TCRs, as has been noted previously.[10] All three lines from well #2 were found to be derived from the same CD8 T cell clone (Figure 1F). Two of the three lines from well #4 seemed to be derived from the same CD8 T cell clone, while the two lines from well #5 represented different clones. Collectively, a total of eight T-iPSC clones bearing different TCRs were established.

T-iPSC Clones Are Heterogeneous in Terms of T Cell-Generating Potential and TCR Affinity

The affinity of TCRs can be speculated by the binding intensity to the tetramer, measured as MFI (mean florescence intensity) by flow cytometry.[11] On the other hand, it is also known that expression of CD8 influences tetramer biding and thus MFI.[12,13] In the case of T-iPSCs, when they are induced to differentiate into the T cell lineage, the generated cells come to express TCR at the $CD4^{-}CD8^{-}$ double-negative (DN) stage.[4] Taking advantage of this finding, we measured tetramer biding intensity of eight clones by analyzing the regenerated cells harvested on day 29 (Figures 2A and 2B), when all cells were still at the DN stage. Tetramer binding intensity compensated by CD3 expression level is also shown (Figure 2C). The results demonstrated that the TCR affinity of the eight cloned T-iPSC lines varies greatly.

We then assessed T cell-generating potential of the eight clones. The most critical point of T cell production from T-iPSC is the frequency of $CD4^{+}CD8^{+}$ double-positive (DP) cells; once DP cells are generated,

it is easy to induce CD8αβ single-positive (SP) cells simply by stimulating the isolated DP cells.[4] Hence, we continued cultivation, and on day 37 we found that DP cells were generated in all cultures (Figure 2D). However, the proportion of DP cells (Figure 2E), as well as the cell yield (Figure 2F), showed a wide range of variation.

One of the aims of this study is to determine the proportion of "usable" T-iPSC clones. We established an arbitrary cutoff for cell yield at 1×10^5 (Figure 2F) and, by this criterion, the top five clones (2-10, 4-1, 4-3, 5-7, 5-10) were considered to be usable in terms of T cell-generating potential. In addition to the results reported here, we have produced ten T-iPSC clones targeting a different antigen (WT1), as well as eleven iPSC clones derived from monocytes, and have evaluated their T cell generating potential (Figures S1A and S1B). We have summarized here the number of initial clones and usable ones in these experiments (Figure 2G). Based on these results, it seems safe to say that about half of T-iPSC clones are usable in terms of their T cell potential.

Antigen-Specific Cytotoxic Activities Were Almost Equivalent Among CTL Clones Bearing TCRs of Different Affinity

We then tested whether functional avidity of regenerated CTLs also varies among clones. Functional avidity was measured as cytotoxic activity against antigen-loaded target cells. For this aim, we selected three T-iPSC clones representing high (2-10), medium (5-7), and low (5-10) TCR affinity (Figures 2B and 2C). For the selection of a representative "medium" clone, clone 4-1, exhibiting highest DP proportion (Figures 2D and 2E), was also considered as one of candidates, but we selected clone 5-7 based on the cell yield data (Figure 2F). After CD8αβ SP cells were induced from DP cells, they were expanded by co-culturing with LCLs loaded with peptide every 7 to 14 days and used as regenerated CTLs in the subsequent experiments.

The regenerated CTLs of the three clones were virtually indistinguishable in terms of $CD4^-CD8^+$ phenotype, as well as exclusive expression of CD8αβ heterodimer (Figure 3A). They were also almost identical for other surface markers associated with the CTL lineage (Figure S2). On the other hand, the MFI of tetramer staining of these regenerated CTLs was different (Figure 3B), reflecting differences in TCR affinity observed when measured on DN cells (Figure 2B). The difference in TCR affinity of the three clones measured on DN cells was not so apparent when measured on regenerated CTLs, probably because of expression of CD8αβ, as mentioned above.

We then addressed the issue whether the regenerated CTLs represent a clonal population or not. While it is possible that some of developing T cells undergo secondary rearrangement and lose the original specificity, it is expected that regenerated CTLs monoclonally express MART-1-specific TCR, since they were expanded by using a specific peptide. Indeed, virtually all cells were found to be MART-1-tetramer-positive for clone 2-10 and clone 5-7, but a portion of cells (∼9%) look tetramer-low or -negative for clone 5-10 (Figure 3B). To investigate clonality of the regenerated CTLs, we served CTLs of these three clones for repertoire analysis (Figure S3). We found that each of three clones exhibited almost complete monoclonality for both TCRα and β chain gene usage. Therefore, it is probable that the tetramer-low/negative cells observed in clone 5-10 are attributed to the failure of tetramer binding due to the low affinity of TCR.

We then assessed cytotoxic activity of regenerated CTLs against peptide-loaded T2 cells or Mel624 cells expressing endogenous MART-1 antigen. No significant difference was observed in both cases when peptide-loaded T2 cells or Mel624 cells were targeted (Figures 3C and 3D). These results indicate that the regenerated CTLs are potent when their TCR affinity is relatively low.

A Low Frequency of Regenerated CTLs Exhibit Alloreactivity

Assuming that regenerated CTLs will be administered in an allogeneic setting to HLA-mismatched recipients, we finally assessed the frequency of occurrence of alloreactivity by using the mixed lymphocyte reaction (MLR). To this end, we prepared a total of 10 LCL lines from 10 healthy volunteers as stimulator cells from putative recipients (Figure 4A). As effector cells, we used four regenerated CTL clones, 2-10, 4-1, 5-7, and 5-10 expressing different TCRs, all MART-1-specific and HLA-A*02:01 restricted. The regenerated CTLs and LCLs were co-cultured for 4 h and the proportion of activated CTLs was measured by gating on CD107a-expressing cells (Figure 4B). The HLA-class I haplotypes in putative donor and recipient cells is shown in Figure 4C. While some recipient alleles are identical to the donor CTL, as indicated in green boxes, a variety of mismatched alleles are present. More precisely, each CTL clone was tested against a total of 26 different HLA-class I alleles.

Using four clones of effector cells and ten lines of stimulator cells, a total of 40 MLR assays were performed. Only one CTL clone, 4-1, showed weak alloreactivity and such alloreactivity was seen against three LCL lines (Figure 4D), which were found to share a common HLA-A*02:06 allele. We further tested alloreactivity of one clone specific to another antigen, WT1, against the same 10 LCL lines, and found that no reaction was detected (Figure S4). In aggregate, the results presented here indicate that the frequency of alloreactivity will be fairly low when CTLs regenerated from T-iPSCs are used in an allogeneic setting.

DISCUSSION

Previous studies including ours have shown that it is possible to expand antigen-specific T cells using iPSC technology.[3,4,14,15] The goal of the present study was to assess whether such a strategy is feasible or not. We established eight T-iPSC clones and found that five of them were able to produce T cells fairly well. The results collectively indicated that the T-iPSC strategy is feasible.

TCR affinity assessed by tetramer binding intensity greatly varied among the regenerated CTL clones. Rather unexpectedly, the three tested clones representing high, medium, and low TCR affinity showed comparable cytotoxic activity against peptide-loaded target

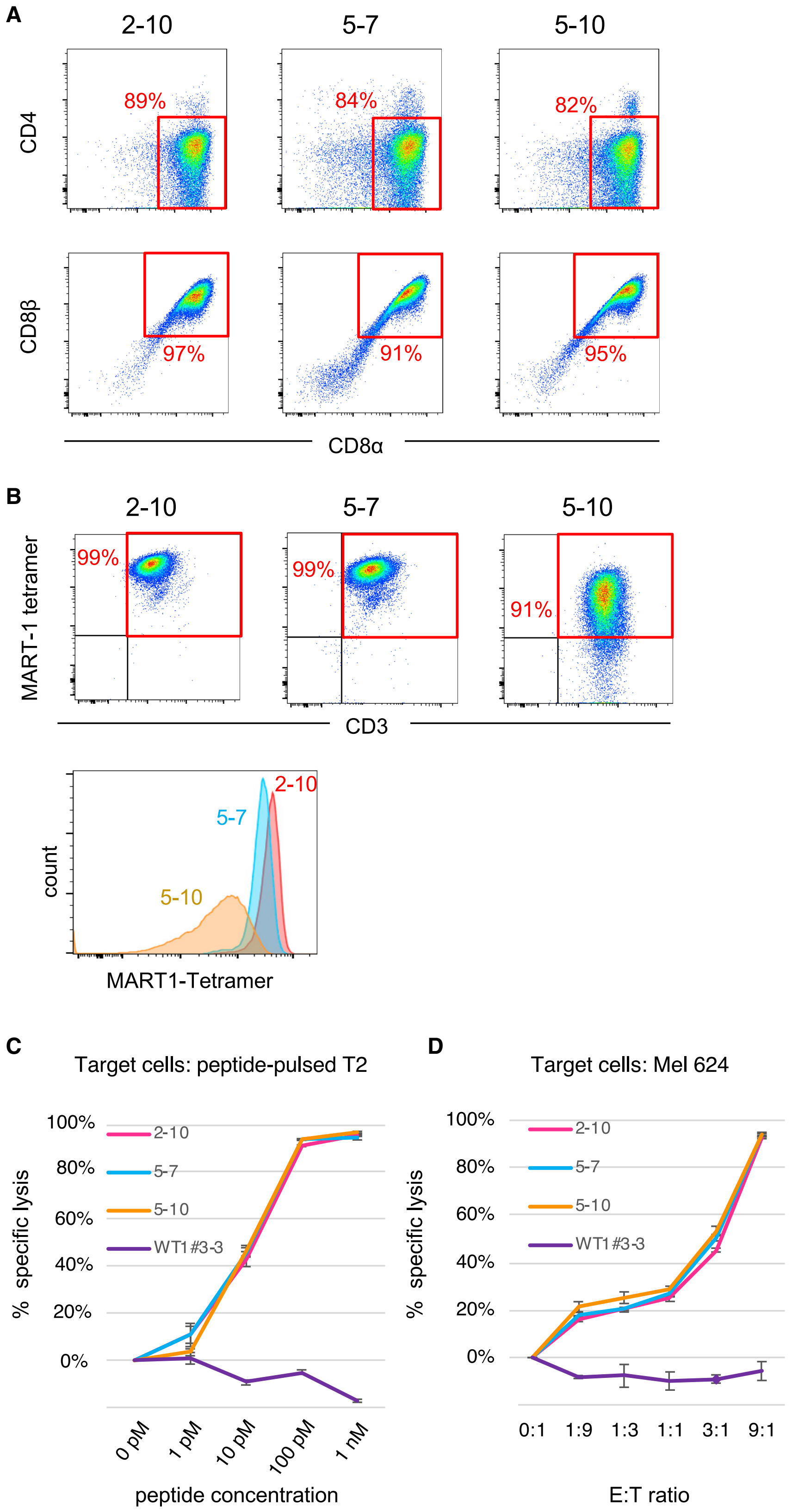

Figure 3. Regenerated CTL Clones Expressing High, Medium, and Low Affinity TCRs Had Almost Equivalent Cytotoxic Activity

(A) Flow cytometric profiles of regenerated CTL clones representing high (2-10), medium (5-7), and low (5-10) TCR affinity stained for CD4, CD8β, and CD8α. (B) Flow cytometric profiles of CTL clones generated on day 29 stained for MART-1-tetramer binding and CD3 expression. (C) An *in vitro* cytotoxic assay was performed by co-culturing each regenerated CTL clone with luciferase-transduced T2 cells (HLA-A02$^+$) loaded with increasing amounts of peptide. The E:T ratio was fixed at 1:1. Living T2 cells at the point with no peptide was regarded as maximal luciferase expression. (D) Cytotoxicity against luciferase-transduced Mel624 cells (HLA-A02$^+$, MART-1$^+$) was measured by different E:T ratio. The cell number of target cells was fixed in each point. Living Mel624 cells at 0:1 point was regarded as maximal luciferase expression. In (C) and (D) CTLs derived from WT1#3-3 T-iPSC (HLA-A24-restricted/WT1-specific) were used as a negative control. Percent lysis was determined as (1 − sample/max) × 100. Data are from one experiment representative of three independent experiments.

A, B

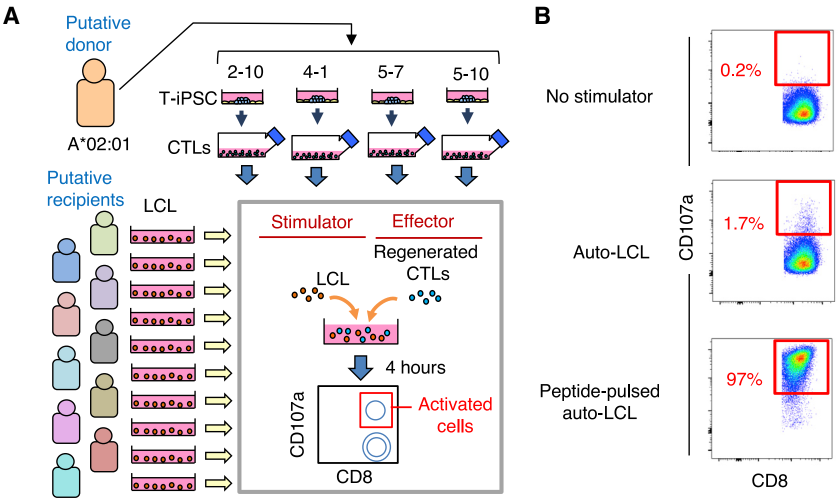

C

	HLA-A	HLA-B	HLA-C
CTL	02:01	15:01	03:03
	24:02	15:11	08:01
LCL001	24:02	52:01	12:02
	31:01	54:01	14:02
LCL002	02:06	40:02	03:04
	11:01		
LCL003	02:10	07:02	07:02
	24:02	40:06	08:01
LCL004	02:06	51:01	14:02
	24:02		15:02
LCL005	31:01	44:03	04:01
	33:03	48:01	14:03
LCL006	02:06	39:01	07:02
	24:02	40:02	15:02
LCL007	26:01	35:01	07:02
	31:01	51:01	14:02
LCL008	02:01	40:06	08:01
	02:10	44:03	14:03
LCL009	02:01	44:03	16:01
LCL010	01:01	15:01	03:03
	02:01	40:01	04:01

D

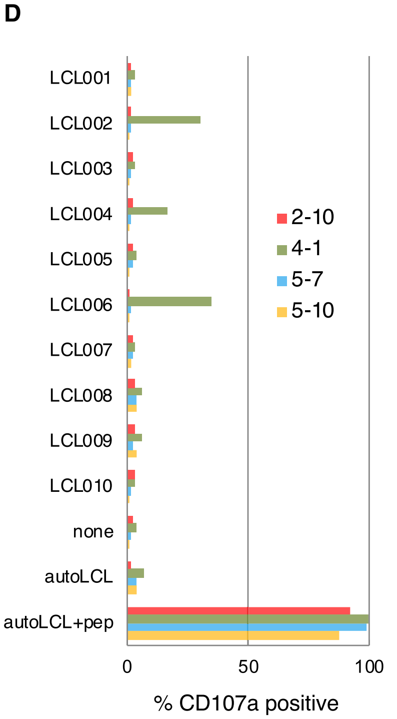

Figure 4. Regenerated CTLs Exhibited Low Frequency Alloreactivity

(A) Schematic illustration of the experimental design to determine the frequency of CTL clones with alloreactivity against allogeneic LCLs. (B) Flow cytometric profile of CTL co-cultured with the indicated cells for 4 h. (C) HLA class I alleles of each LCL used as a stimulator. Green boxes indicate the alleles identical to those of the CTL lines. (D) Bars represent the percentage of CD107a-positive activated cells. The ratio of CTLs/stimulators was fixed at 1:1. Autologous LCLs without peptide pulse were used as a negative control, and autologous LCLs pulsed with MART-1-peptide were used as a positive control. Data are from one experiment representative of two independent experiments.

cells or endogenous MART-1 antigen-expressing cells. This finding may be explained by the process of so-called “avidity maturation,” where TCR avidity is tuned by various mechanisms during immune responses.[16–18] On the other hand, the finding that “CTLs are potent regardless of their TCR affinity” cannot necessarily be generalized, since MART-1 antigen is exceptional in that it is highly immunogenic and that the TCR clones reactive to MART-1 are present in naive T cell populations at much higher frequency compared with other antigens.

An alloreactive response was seen in three cases out of a total of 50 MLR assays (four MART-1-TCRs and one WT1-TCR versus 10 LCLs) that were performed assuming an allogeneic transfer setting. Since it is generally accepted that the frequency of T cell clones that exhibit alloreactivity is around 10%,[19–21] this frequency (3/50) was within expectation. When we consider the frequency of alloreactivity in terms of “one TCR versus one HLA-class I allele,” it can be said that we have tested five TCRs against 26 mismatched class I alleles, i.e., a total of 130 tests. In this context, it was seen that HLA-A*02:06 was expressed in all three LCL lines that induced an allo response by the 4-1 CTL clone, but not in the other LCL lines (Figure 4C). Because HLA-A*02:06 versus HLA-A*02:01 is known as a high risk HLA allele mismatch combination for graft-versus-host disease (GVHD) in hematopoietic stem cell transplantation,[22,23] it is likely that the 4-1 CTL clone had an alloreaction against HLA-A*02:06. In sum, our date demonstrated that only one case in 130 tests of a TCR versus mismatched class I alleles resulted in activation of CTLs, indicating that the risk of alloreactivity when using the T-iPSC method in an allogenic setting is quite low. In other words, we would propose that having one spare T-iPSC clone is sufficient to deal with the case that regenerated CTLs exhibit alloreactivity against recipient cells in an MLR assay performed prior to cell transfer therapy.

In the present study, all eight clones were able to give rise to T cells, and five of them were capable of producing a good number of CTLs. In different experiments using T-iPSCs that targeted the WT1 antigen, as well as iPSCs derived from monocytes, 4 in 10, and 6 in 11 clones, respectively, were found to be usable for regenerating CTLs (Figure 2G). Therefore, we can estimate that about half of T-iPSC clones are capable of robustly regenerating CTLs. Such a high frequency of T-iPSC clones retaining T cell potential may reflect the

fact that they are originally derived from blood cells, since it has been reported that iPSCs retain a tendency to more efficiently produce the tissues from which the iPSCs originated.[24] The frequency of iPSC lines retaining T cell potential would likely be different when different tissue cells are used as a starting material, when a different method is used to produce iPSCs, even if the original material is T cells. The results of the present study clearly show that T-iPSC clones produced by our method retain T cell potential with high frequency (~50%).

Based on our studies, we can now estimate how many clones are initially required to obtain usable T-iPSC clones. Our data demonstrated that roughly 50% of T cell-derived iPSC clones are capable of producing potent CTLs. When we apply this to an autologous setting, one usable clone is enough, since alloreactivity is not an issue. In this case, it can be calculated that one can reliably (>95% probability) obtain one usable clone when five clones are initially produced. When we apply this method to an allogeneic setting, it is necessary to prepare spare clones to deal with cases where regenerated CTLs show alloreactivity against recipient cells in the MLR assay that will be performed before treatment. Since the frequency of CTL clones showing alloreactivity was found to be quite low (Figure 4D), we would propose that preparation of one spare clone is enough. If we wish to reliably obtain two potent clones, how many clones should we initially prepare, provided that an individual clone is good enough with 50% frequency? In this case, by mathematical calculation (see Materials and Methods), it is estimated that eight clones are enough, which is actually a reasonable number.

The efficiency of reprogramming (T cells to iPSCs) was found to be around $1/10^5$ (Table S1). In the present study, starting from 1.8×10^7 PBMC prepared from 20 mL blood, eight T-iPSC clones were established. This efficiency seems good enough, but cannot be generalized, because frequency of MART-1-specific T cells is exceptionally high compared with other antigens, as mentioned earlier. If you wish to clone CTLs as iPSC lines like the present study, in case of antigens of lower frequency, more blood will be required. However, for such antigens, an alternative approach is possible: first you establish a potent CTL clone, and then produce iPSCs from the CTLs of the clone. Since the reprogramming efficiency is $1/10^5$ as described above, when a CTL clone is expanded to 10^6 cells, it is possible to produce ten T-iPSC clones, which is sufficient enough to get two usable T-iPSC clones.

Recently, it was reported that the iPSCs transduced with exogenous TCR gene gave rise to potent CTLs.[25] This TCR-transduction method may make it easier to produce a cell source for CTLs. However, in that paper it remained unclear whether CTLs produced from TCR-transduced iPSCs are as good as those from T-iPSCs in terms of cytotoxic activity, since these two types of CTLs were not directly compared. Moreover, the TCR-iPSC method has the following three concerns: (1) random integration of TCR gene may bring about the risk to damage the genome, (2) it is difficult to control the expression level of TCR, and (3) it will be regarded as a gene therapy, making the regulation issues more difficult. On the other hand, the T-iPSC method is free from these points.

Collectively, we conclude that the strategy to produce T-iPSCs is feasible not only in an autologous setting but also in an allogeneic setting.

MATERIALS AND METHODS

Study Approval

This study was approved by the Institutional Review Board of the Graduate School of Medicine, Kyoto University (approval number: G761) and abided by the tenets of the Declaration of Helsinki. All specimens from healthy individuals were collected after written informed consent was obtained.

Human Subjects

Blood samples were obtained from healthy donors after obtaining informed consent for sample procurement as approved by Kyoto University Hospital. PBMCs from healthy volunteers were isolated using Ficoll-Paque PLUS (GE Healthcare).

Cell Lines

OP9 and OP9-DLL1 cells were maintained in α-MEM supplemented with 20% fetal bovine serum (FBS). The T2 cell line was purchased from ATCC and was maintained in RPMI with 10% FBS. The Mel624 cell line was provided by Dr. Rosenberg (NIH) and maintained in DMEM with 10% FBS. All media contained 1% penicillin-streptomycin. LCLs were established in our laboratory using Epstein-Barr virus produced by B95-8 cell lines to infect and transform human B lymphocytes into LCLs *in vitro*.

Expansion of MART-1-Specific CTLs from Primary PBMCs by Stimulation with Peptide and Peptide-Loaded LCLs

Following collecting blood from an HLA-A*02:01 volunteer, 1.8×10^7 PBMCs were divided into nine lots (2×10^6/lot), and cells of each lot were cultured in eight wells of a 96-well plate in RPMI 1640 medium with 10% human serum and 1% penicillin-streptomycin in the presence of MART-$1_{26\text{-}35(A27L)}$ (ELAGIGILTV) synthetic peptide (final 10 μM) as a priming. After 2 days, recombinant interleukin-2 (IL-2) (12.5 U/mL), IL-7 (10 ng/mL), and IL-21 (30 ng/mL) (Peprotech) were added to each well. 2 weeks later, cells from eight wells were collected and co-cultured in a well of 24-well plates (total nine wells) with 50 Gy irradiated HLA-A*02:01$^+$ LCL loaded with 100 nM MART-1-peptide (T cell: LCL ratio 10:1) as an expansion phase. These T cells were stimulated once per week for further expansion.

Establishment of iPCs Derived from MART-1-Specific T Cells and from Monocyte

iPSCs derived from CD8$^+$ T cells (T-iPSCs) were established by the previously reported method with slight modifications.[3,4] Briefly, after expanding primary CD8 T cells, MART-1-tetramer (HLA-A*02:01 Mart-1 Tetramer-ELAGIGILTV-PE, MBL)-positive CD8 T cells

were sorted (FACSAriaIII, BD Biosciences) and then stimulated with CD3/CD28 magnetic beads (Dynabeads, Thermo Fisher Scientific) in 96-well U bottom plates. 2 days later, cells were transduced with two lines of Sendai virus vector for the transient expression of four Yamanaka factors[26] and SV40 large T antigen (DNAVEC). Following 2 h incubation at 37°C, cells were collected and seeded onto murine embryonic fibroblasts feeder cells and cultured in the same media as used for culturing primary CTLs. After 2 days of cultivation, half of the medium was replaced every day with human iPSC medium (Repro Stem, REPROCELL) supplemented with 5 ng/mL basic fibroblast growth factor (bFGF, Wako). iPSC colonies started to appear from 11–14 days, then we replaced the whole medium every day. Each colony was picked up within days 21–35 and established as iPSC clones.

As for the establishment of iPSCs derived from monocyte (non-T-iPSCs), 1×10^6 monocytes were enriched by depletion of $CD3^+CD19^+$ cells followed by positive separation of $CD14^+$ cells with magnetic beads (MACS, Milteny Biotec), and then they were transduced with two lines of Sendai virus vector as described above.

Differentiation of T Cells from T-iPSCs

T-iPSCs were differentiated to $CD4^+CD8^+$DP cells using OP9 and OP9-DLL1 stromal cell co-culture systems as previously described with slight modifications.[3,4] In brief, iPSC colonies were dissociated using trypsin (0.25%) and collagenase IV (1 mg/mL) and mechanically disrupted into small clumps by pipetting. About 600 iPSC clumps were collected and plated on gelatin pre-coated OP9 dishes filled with OP9 medium (α-MEM, Invitrogen, with 20% FCS). On day 13, colonies containing $CD34^+$ progenitor cells were treated with collagenase type IV (50 U/mL) and trypsin-EDTA (0.05%). These collected cells were plated on an OP9-DLL1 semi-confluent dish with OP9 medium containing hIL-7 (5 ng/mL), hFlt-3L (5 ng/mL), and hSCF (5 ng/mL) (Peprotech) for differentiation into T cell progenitors. On day 18, floating cells were collected and transferred into a new dish layered with OP9-DLL1. From this point, these exchange procedures to new feeder cells were done every week. On days 36–40, floating cells were collected and $CD4^+CD8^+$DP cells were enriched using anti-CD4 microbeads (Milteny Biotec). These isolated cells were stimulated every 7 days with 50 Gy irradiated HLA-A*02:01$^+$ LCL loaded with 100 nM MART-1-peptide at a 2:1 of T cell/LCL ratio with 20% FBS α-MEM medium in the presence of hIL-7 (5 ng/mL) and hIL-21 (10 ng/mL) (Peprotech). The obtained CD8αβ type regenerated CTLs were stimulated at a 5:1 ratio for further expansion.

TCR Sequence

MART-1 specificity of each regenerated $CD4^-CD8^-$DN T cells at day 30 was confirmed by flow cytometry and then tetramer-positive cells were sorted by FACS AriaIII. Total RNA was extracted from each regenerated MART-1-specific T cell clone (RNA Plus Mini Kit, QIAGEN) and cDNA was synthesized by using SuperScript VILO cDNA Synthesis kit (Thermo Fisher). Then nested PCR was performed according to the manufacturer's instructions (SMARTer RACE cDNA Amplification Kit, Clontech). By using amplified DNA samples, DNA sequencing was performed. The IMGT/V-QUEST search engine was used for determining the identity of each TCR gene (http://www.imgt.org/IMGT_vquest/vquest).

Cytotoxicity Assays *In Vitro* Using Luminescence

The cytotoxicity of regenerated CTLs was determined by a standard luciferase-based assay. Briefly, T2 (MART-1$^-$, HLA-A02$^+$) and Mel624 (MART-1$^+$, HLA-A02$^+$) cells transduced with luciferase-ZsG (pHIV-Luc-ZsGreen, Addgene) served as target cells. 3×10^4 target cells were seeded on a well of 96-well plates. The effector cells (E), i.e., regenerated CTLs and target cells (T), were co-cultured in triplicate at the different E/T ratio in the presence of hIL-7 (2.5 ng/mL). 15–18 h later, 100 μL of luciferase substrate (Bright-Glo, Promega) was added to each well, and emitted light was detected with black-walled 96-well plates in a luminescence plate reader (GloMax Explorer, Promega).

HLA Typing

HLA typing was performed at the HLA Foundation Laboratory (Kyoto, Japan), with PCR-rSSO using WAKFlow (Wakunaga Pharmaceutical).

CD107a Assay to Detect Alloreactivity

1×10^5 cells of each regenerated CTLs were co-cultured with each target allo-LCL clone at a 1:1 E/T ratio using 96-well plates in the presence of BD GolgiStop (1:3,000 dilution) (BD Biosciences) and anti-CD107a (1:200 dilution). 4 h later, cells were collected and flow cytometry analysis was performed following staining with anti-CD8 to gate on the T cells.

Flow Cytometry

The following monoclonal antibodies (clone name) were used: CD3 (UCHT-1), CD4 (RPA-T4), CD8α (RPA-T8, HIT8a), CD8β (2ST8.5H7), CD5 (UCHT-2), CD2 (RPA-2.10), LAG-3 (17B4, Enzo Life Sciences), CTLA-4 (L3D10), PD-1 (EH12.2H7), CD27 (M-T271), CD28 (CD28.2), CD45RA (HI100), CD45RO (UCHL1), CD62L (DREG-56), CCR7 (150503), NKG2C (FAB138G-025, R&D Systems), NKG2D (1D11), DNAM-1 (11A8), CD56 (HCD56), NKp30 (P30-15), NKp44 (P44-8), NKp46 (9E2), and CD107a (H4A3). All Abs were purchased from BioLegend or BD Biosciences except as indicated above. HLA-A*02:01 MART-1 tetramer, ELAGIGILTV-PE (MBL) was used for the detection of T cells expressing TCR specific for the peptide binding to HLA-A*02:01. Flow cytometric analysis was performed using a FACS CantoII or FACS AriaIII (BD Biosciences). FACS data was analyzed by FlowJo software (BD Biosciences).

Calculation of Probabilities of Obtaining a Potent T-iPSC Clone

If we would like to have N = 1 potent clone out of M clones, it is successful unless all M clones are unusable, which takes place with a $1/2^M$chance based on our premise that half of the T-iPSC clones are usable. Therefore, we find M = 5 by solving for the smallest M that satisfies $1\text{-}1/2^M > 0.95$ or $0.05 > 1/2^M$. Similarly, if we

would like to have N = 2 potent clones out of M clones, it is successful unless all M clones are unusable (a chance of $1/2^M$) or only one clone is usable (a chance of $M \times 1/2^M$). Therefore, we find M = 8 by solving for the smallest M that satisfies $1 - (1 + M) \times 1/2^M > 0.95$ or $0.05 > (1 + M) \times 1/2^M$. In general, the probability of getting more than N potent clones from M clones is calculated as shown:

$$\frac{1}{2^M}\sum_{\{k=N\}}^{M}\binom{M}{k} = 1 - \frac{1}{2^M}\sum_{\{k=0\}}^{N-1}\binom{M}{k}.$$

If we wish to obtain N = 1, 2, 3, ... usable clones with >95% probability, we need, respectively, to initially establish M = 5, 8, 11, ... clones.

TCR Repertoire Analysis of Regenerated CTLs

TCR repertoire analysis of regenerated CTLs was perfomed by next-generation sequencing. Each RNA from three representative reCTLs was extracted with ISOGEN (Nippon Gene). NGS was performed with an unbiased TCR repertoire analysis developed by Repertoire Genesis (Osaka, Japan) in which an unbiased adaptor-ligation PCR was performed as described previously.[27] Bioinformatics analysis was then performed by Repertoire Genesis.

SUPPLEMENTAL INFORMATION

Supplemental Information can be found online at https://doi.org/10.1016/j.omtm.2019.12.006.

AUTHOR CONTRIBUTIONS

S.N. and T.M. performed the experiments and analyzed the data. T.M., H.I., and S.K. contributed to development of experiment methods. M.O., M.N., T.K., N.K., and K.M. gave technical advice in performing the experiments. A.T.-K. and H.K. designed the project. S.N., K.M., and H.K. interpreted data and wrote the manuscript.

ACKNOWLEDGMENTS

This work was supported by P-Direct (Project for the Development of Innovative Research on Cancer Therapeutics) from the Ministry of Education, Culture, Sports, Science and Technology, Japan, by Astlym Co. Ltd and by RegCell Co. Ltd. We thank Shizuo Kaji (Kyushu University) for advising about calculating probabilities and Peter Burrows (University of Alabama at Birmingham) for critical reading of the manuscript.

REFERENCES

1. Rosenberg, S.A., and Restifo, N.P. (2015). Adoptive cell transfer as personalized immunotherapy for human cancer. Science *348*, 62–68.
2. June, C.H., O'Connor, R.S., Kawalekar, O.U., Ghassemi, S., and Milone, M.C. (2018). CAR T cell immunotherapy for human cancer. Science *359*, 1361–1365.
3. Vizcardo, R., Masuda, K., Yamada, D., Ikawa, T., Shimizu, K., Fujii, S., Koseki, H., and Kawamoto, H. (2013). Regeneration of human tumor antigen-specific T cells from iPSCs derived from mature CD8(+) T cells. Cell Stem Cell *12*, 31–36.
4. Maeda, T., Nagano, S., Ichise, H., Kataoka, K., Yamada, D., Ogawa, S., Koseki, H., Kitawaki, T., Kadowaki, N., Takaori-Kondo, A., et al. (2016). Regeneration of CD8αβ T cells from T-cell-derived iPSC imparts potent tumor antigen-specific cytotoxicity. Cancer Res. *76*, 6839–6850.
5. Sugita, S., Iwasaki, Y., Makabe, K., Kimura, T., Futagami, T., Suegami, S., and Takahashi, M. (2016). Lack of T cell response to iPSC-derived retinal pigment epithelial cells from HLA homozygous donors. Stem Cell Reports *7*, 619–634.
6. Cahan, P., and Daley, G.Q. (2013). Origins and implications of pluripotent stem cell variability and heterogeneity. Nat. Rev. Mol. Cell Biol. *14*, 357–368.
7. Yee, C., Savage, P.A., Lee, P.P., Davis, M.M., and Greenberg, P.D. (1999). Isolation of high avidity melanoma-reactive CTL from heterogeneous populations using peptide-MHC tetramers. J. Immunol. *162*, 2227–2234.
8. Ochi, T., Nakatsugawa, M., Chamoto, K., Tanaka, S., Yamashita, Y., Guo, T., Fujiwara, H., Yasukawa, M., Butler, M.O., and Hirano, N. (2015). Optimization of T-cell reactivity by exploiting TCR chain centricity for the purpose of safe and effective antitumor TCR gene therapy. Cancer Immunol. Res. *3*, 1070–1081.
9. Pittet, M.J., Valmori, D., Dunbar, P.R., Speiser, D.E., Liénard, D., Lejeune, F., Fleischhauer, K., Cerundolo, V., Cerottini, J.C., and Romero, P. (1999). High frequencies of naive Melan-A/MART-1-specific CD8(+) T cells in a large proportion of human histocompatibility leukocyte antigen (HLA)-A2 individuals. J. Exp. Med. *190*, 705–715.
10. Pinto, S., Sommermeyer, D., Michel, C., Wilde, S., Schendel, D., Uckert, W., Blankenstein, T., and Kyewski, B. (2014). Misinitiation of intrathymic MART-1 transcription and biased TCR usage explain the high frequency of MART-1-specific T cells. Eur. J. Immunol. *44*, 2811–2821.
11. Gallegos, A.M., Xiong, H., Leiner, I.M., Sušac, B., Glickman, M.S., Pamer, E.G., and van Heijst, J.W. (2016). Control of T cell antigen reactivity via programmed TCR downregulation. Nat. Immunol. *17*, 379–386.
12. Daniels, M.A., and Jameson, S.C. (2000). Critical role for CD8 in T cell receptor binding and activation by peptide/major histocompatibility complex multimers. J. Exp. Med. *191*, 335–346.
13. Laugel, B., van den Berg, H.A., Gostick, E., Cole, D.K., Wooldridge, L., Boulter, J., Milicic, A., Price, D.A., and Sewell, A.K. (2007). Different T cell receptor affinity thresholds and CD8 coreceptor dependence govern cytotoxic T lymphocyte activation and tetramer binding properties. J. Biol. Chem. *282*, 23799–23810.
14. Nishimura, T., Kaneko, S., Kawana-Tachikawa, A., Tajima, Y., Goto, H., Zhu, D., Nakayama-Hosoya, K., Iriguchi, S., Uemura, Y., Shimizu, T., et al. (2013). Generation of rejuvenated antigen-specific T cells by reprogramming to pluripotency and redifferentiation. Cell Stem Cell *12*, 114–126.
15. Ando, M., Nishimura, T., Yamazaki, S., Yamaguchi, T., Kawana-Tachikawa, A., Hayama, T., Nakauchi, Y., Ando, J., Ota, Y., Takahashi, S., et al. (2015). A safeguard system for induced pluripotent stem cell-derived rejuvenated T cell therapy. Stem Cell Reports *5*, 597–608.
16. Valitutti, S., Müller, S., Dessing, M., and Lanzavecchia, A. (1996). Signal extinction and T cell repolarization in T helper cell-antigen-presenting cell conjugates. Eur. J. Immunol. *26*, 2012–2016.
17. Slifka, M.K., and Whitton, J.L. (2001). Functional avidity maturation of CD8(+) T cells without selection of higher affinity TCR. Nat. Immunol. *2*, 711–717.
18. Fahmy, T.M., Bieler, J.G., Edidin, M., and Schneck, J.P. (2001). Increased TCR avidity after T cell activation: a mechanism for sensing low-density antigen. Immunity *14*, 135–143.
19. Lindahl, K.F., and Wilson, D.B. (1977). Histocompatibility antigen-activated cytotoxic T lymphocytes. II. Estimates of the frequency and specificity of precursors. J. Exp. Med. *145*, 508–522.
20. Suchin, E.J., Langmuir, P.B., Palmer, E., Sayegh, M.H., Wells, A.D., and Turka, L.A. (2001). Quantifying the frequency of alloreactive T cells in vivo: new answers to an old question. J. Immunol. *166*, 973–981.
21. Macedo, C., Orkis, E.A., Popescu, I., Elinoff, B.D., Zeevi, A., Shapiro, R., Lakkis, F.G., and Metes, D. (2009). Contribution of naïve and memory T-cell populations to the human alloimmune response. Am. J. Transplant. *9*, 2057–2066.
22. Kawase, T., Morishima, Y., Matsuo, K., Kashiwase, K., Inoko, H., Saji, H., Kato, S., Juji, T., Kodera, Y., and Sasazuki, T.; Japan Marrow Donor Program (2007). High-risk HLA allele mismatch combinations responsible for severe acute graft-versus-host disease and implication for its molecular mechanism. Blood *110*, 2235–2241.

23. Hwang, S.H., Oh, H.B., Choi, S.E., Seo, J.J., Lee, J.H., Cho, S.W., Chae, J.M., Heo, Y.S., Chang, C.L., and Lee, E.Y. (2010). Impact of amino acid substitution at residue 9 of HLA-A2 on the development of acute GVHD in Korean pediatric patients receiving unrelated hematopoietic stem cell transplantation. Transpl. Int. *23*, 1216–1222.

24. Kim, K., Doi, A., Wen, B., Ng, K., Zhao, R., Cahan, P., Kim, J., Aryee, M.J., Ji, H., Ehrlich, L.I., et al. (2010). Epigenetic memory in induced pluripotent stem cells. Nature *467*, 285–290.

25. Minagawa, A., Yoshikawa, T., Yasukawa, M., Hotta, A., Kunitomo, M., Iriguchi, S., Takiguchi, M., Kassai, Y., Imai, E., Yasui, Y., et al. (2018). Enhancing T cell receptor stability in rejuvenated iPSC-derived T cells improves their use in cancer immunotherapy. Cell Stem Cell *23*, 850–858.e4.

26. Nishimura, K., Sano, M., Ohtaka, M., Furuta, B., Umemura, Y., Nakajima, Y., Ikehara, Y., Kobayashi, T., Segawa, H., Takayasu, S., et al. (2011). Development of defective and persistent Sendai virus vector: a unique gene delivery/expression system ideal for cell reprogramming. J. Biol. Chem. *286*, 4760–4771.

27. Yoshida, R., Yoshioka, T., Yamane, S., Matsutani, T., Toyosaki-Maeda, T., Tsuruta, Y., and Suzuki, R. (2000). A new method for quantitative analysis of the mouse T-cell receptor V region repertoires: comparison of repertoires among strains. Immunogenetics *52*, 35–45.

OMTM, Volume *16*

Supplemental Information

High Frequency Production of T Cell-Derived iPSC Clones Capable of Generating Potent Cytotoxic T Cells

Seiji Nagano, Takuya Maeda, Hiroshi Ichise, Soki Kashima, Manami Ohtaka, Mahito Nakanishi, Toshio Kitawaki, Norimitsu Kadowaki, Akifumi Takaori-Kondo, Kyoko Masuda, and Hiroshi Kawamoto

Well #	Sorter count	Manual count after sorting	No. of initial iPSC-like colonies	No. of established iPSC clones
1	1.65×10^5	N.D	4	1
2	4.03×10^5	N.D	8	3
3	3.14×10^5	N.D	0	0
4	0.99×10^5	N.D	3	3
5	2.65×10^5	N.D	5	2
6	1.45×10^5	N.D	1	0
7	3.16×10^5	2.16×10^5	5	1
8	1.41×10^5	0.8×10^5	4	1
9	N.D			
Total	1.84×10^6	1.1×10^6 (estimated)	30	11

Table S1. Efficiencies of establishing T-iPSC clones from MART-1 specific CD8 T cells

Numbers of MART-1$^+$CD8$^+$cells isolated by a cell sorter are shown as a sorter count and a manual count, as well as numbers of iPSC-like colonies initially formed and eventually established as iPSC clones, are displayed for each well in the experiment shown in Figure 1.

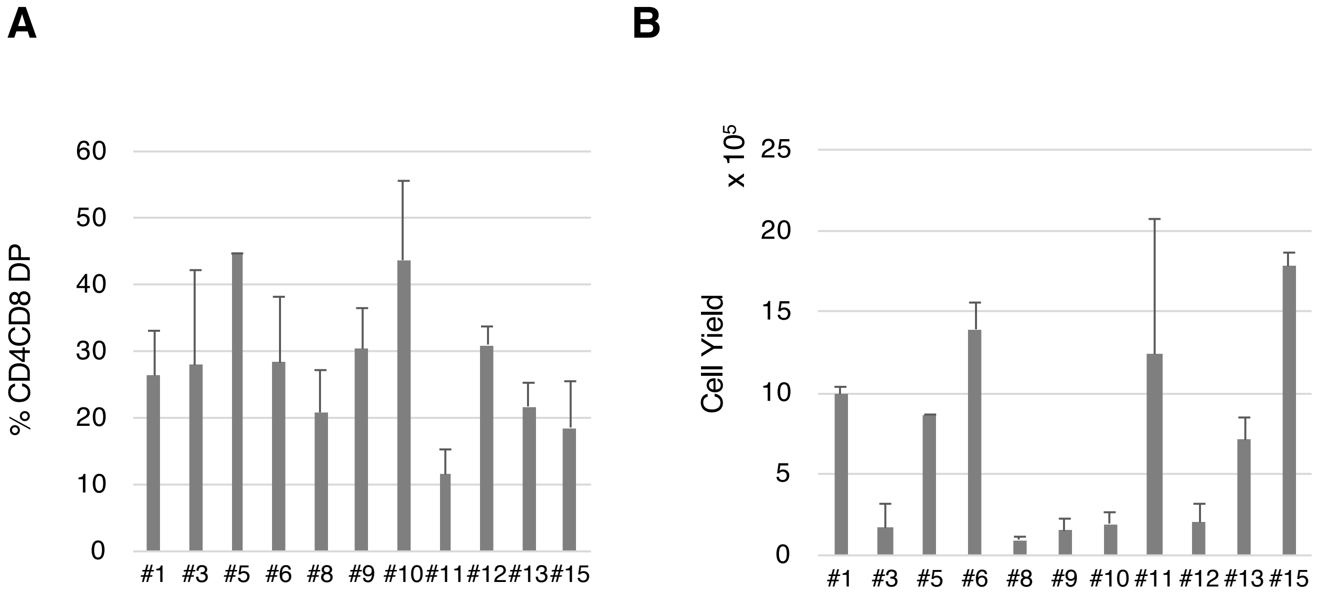

Figure S1. Heterogeneity of monocyte-derived non-T-iPSC clones with regards to T cell generating potential

A) The percentage of $CD4^{+}CD8^{+}$DP cells in cells generated from each non-T-iPSC clone. **B)** The total number of cells generated from each clone in one 10 cm dish. Data are shown as averages of two or three independent experiments with error bars representing SEM.

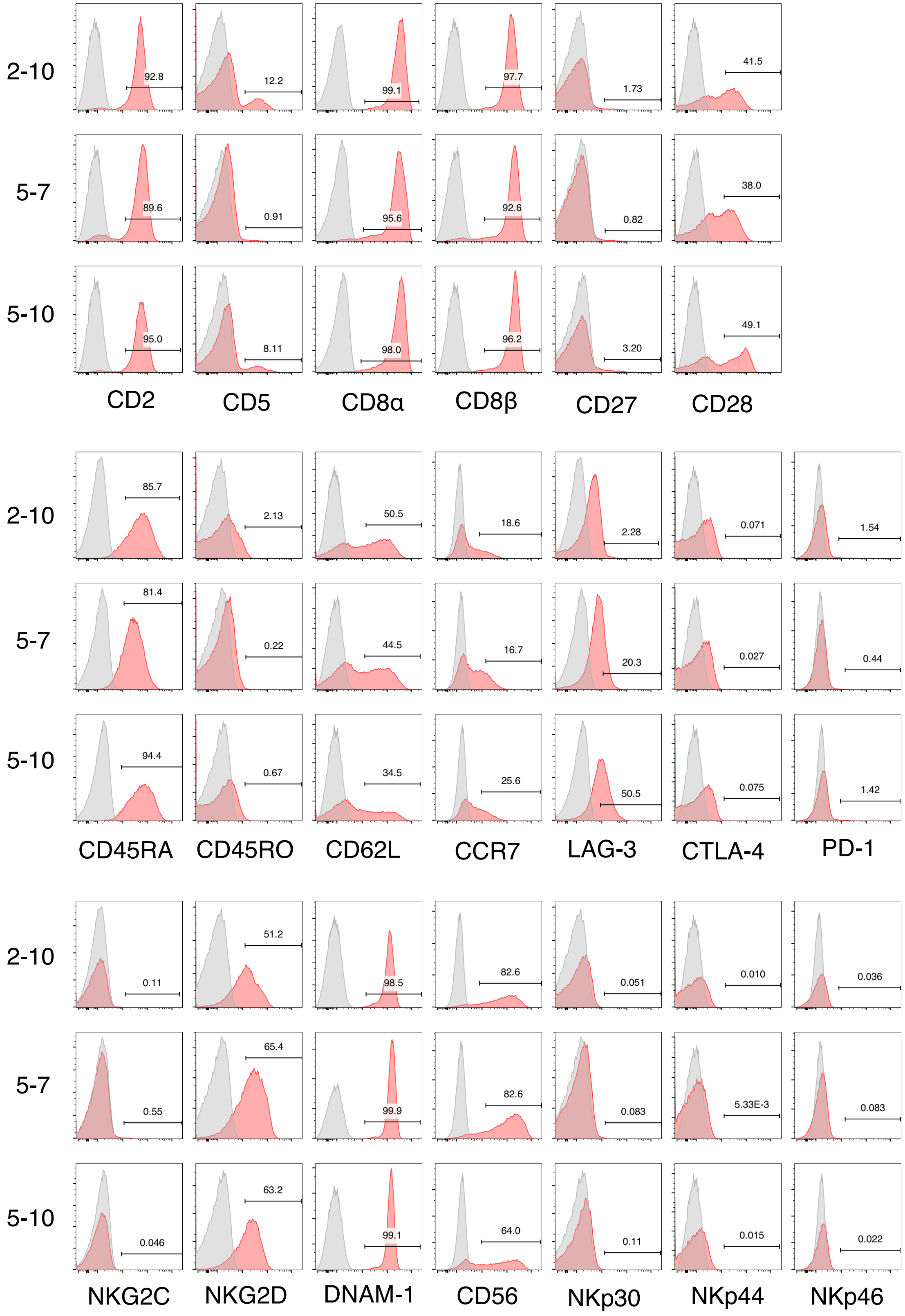

Figure S2. The regenerated CTL clones showed almost identical cell surface phenotypes

Flow cytometric analysis of three regenerated CTL clones representing TCR high (2-10), medium (5-7) and low (5-10) affinity, for the expression of indicated antigens.

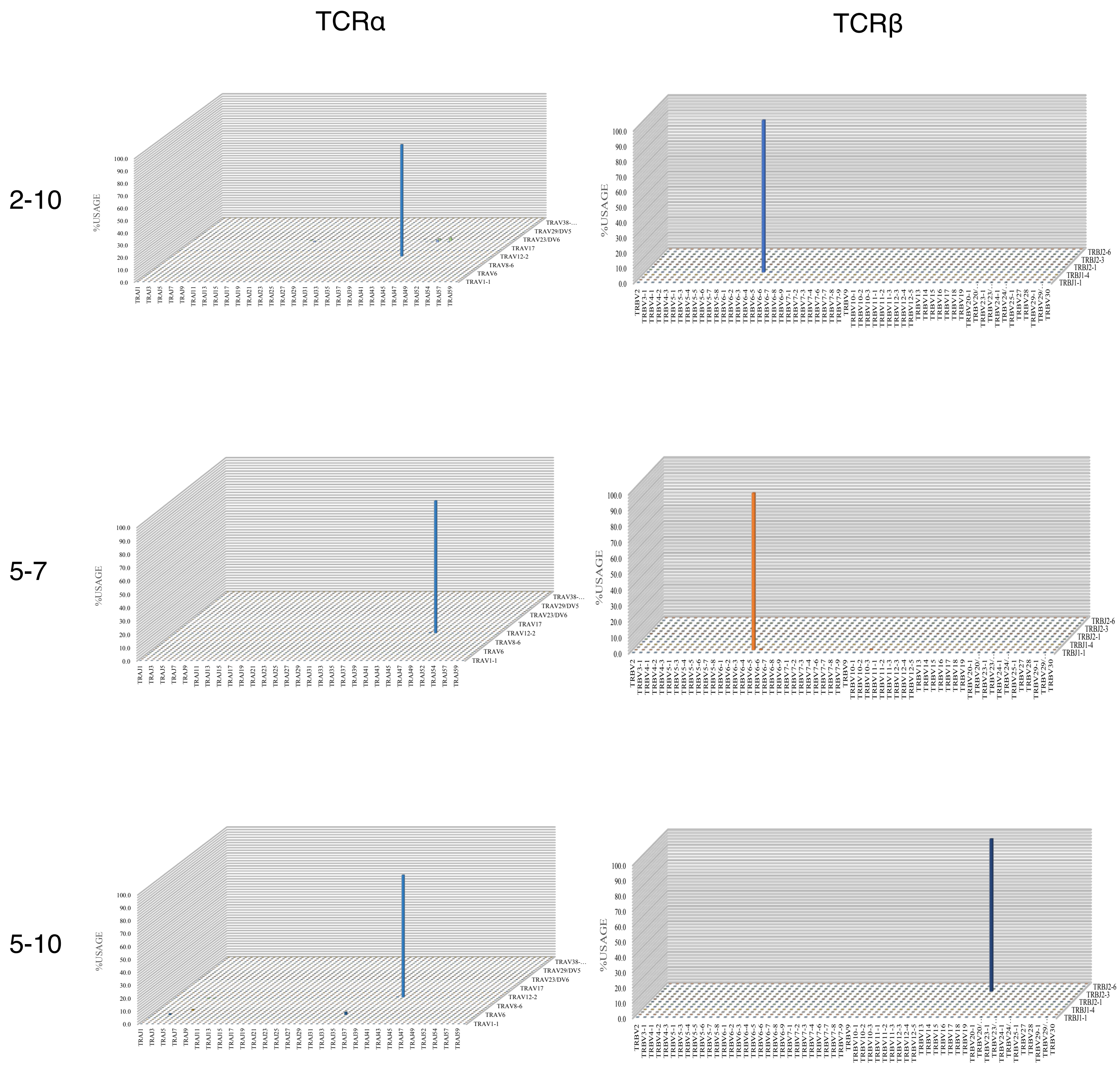

Figure S3. TCR repertoire analysis of regenerated CTLs demonstrates their monoclonality

CTLs regenerated from representative three T-iPSC clones (2-10, 5-7, 5-10) were analyzed for specific CDR3 amino acid sequences, usages of TCRα chain variable (TRAV) and joining (TRAJ) region genes for TCRα, as well as usages of TCRβ chain variable (TRBV) and joining (TRBJ) region genes for TCRβ genes.

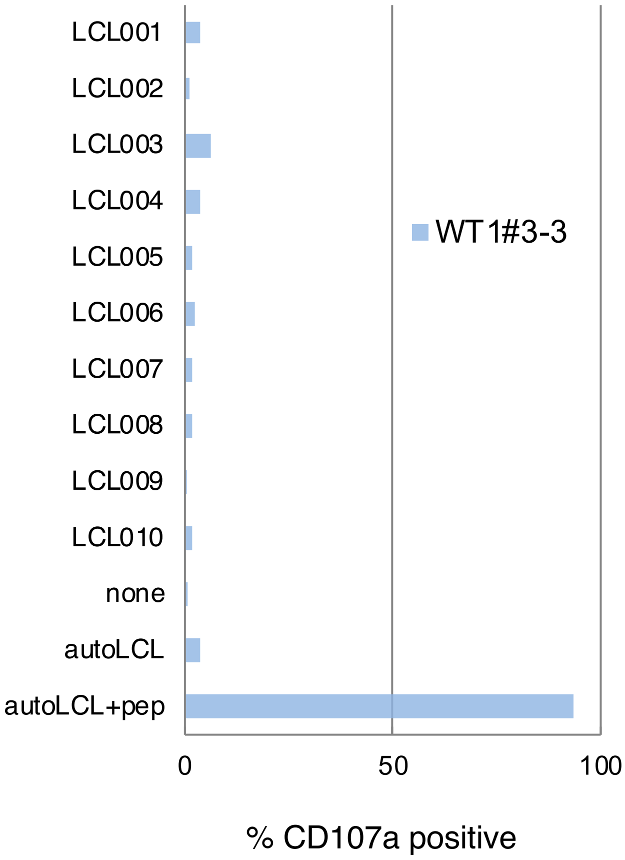

Figure S4. Regenerated CTLs specific for WT1 antigen did not show alloreactivity against ten LCLs

Regenerated CTLs from WT1#3-3 T-iPSCs were tested for allo-reactivity in the same manner as Figure 4. Bars represent the percentage of CD107a positive activated cells. The ratio of CTLs/stimulators was fixed at 1:1. Autologous LCL without peptide pulse was used as a negative control, and autologous LCL pulsed with WT1-peptide$_{235\text{-}243}$ was used as a positive control.
